# Transcatheter Closure of Partially Ligated Vertical Vein after Surgical Correction of Supracardiac Total Anomalous Pulmonary Venous Connection

**Published:** 2015-07-03

**Authors:** Hamid Amoozgar, Maryam Ahmadipoor, Ahmad Ali Amirghofran

**Affiliations:** 1 *Neonatology and Cardiac Research Center, Shiraz University of Medical Sciences, Shiraz, Iran.*; 2 *Cardiac Surgery Department, Shiraz University of Medical Sciences, Shiraz, Iran.*

**Keywords:** *Cardiac catheterization*, *Pulmonary veins*, *Septal occluder device*

## Abstract

Total anomalous pulmonary venous connection (TAPVC) is an anomaly in which the pulmonary veins are directly connected to one of the systemic veins or drain into the right atrium. Management of pulmonary hypertension after the total correction of this congenital cardiac anomaly is very important. Unligation of the vertical vein in the supracardiac type of this anomaly can be a draining pathway for the prevention of postoperative pulmonary hypertension crisis. Late onset transcatheter closure of the unligated vertical vein after a decrease in pulmonary pressure with the Amplatzer vascular plug type 1can prevent residual left-to-right shunting. Here we describe two patients who previously underwent surgical correction of supracardiac TAPVC and their vertical veinwas partially ligated due to severe pulmonary hypertension. Consequently, because of increased left-to-right shunting in the follow-up period, transcatheter occlusion of the vertical vein was done for them and this procedure seemed safe and less invasive compared to the surgical approach.At 2 years'follow-up, there was marked pulmonary artery pressure and clinical improvement.

## Introduction

Total anomalous pulmonary venous connection (TAPVC) is definedas an anomaly in which the pulmonary veins have no connection with the left atrium. Rather, the pulmonary veins are directly connected to one of the systemic veins or drain into the right atrium.^1^

The frequency of TAPVC is between 1-1.5% of all congenital heart diseases and it can be associated with other cardiac anomalies, especially the heterotaxysyndrome.^2^ The signs and symptoms of TAPVC are variable depending on the site of the drainage of the pulmonary veins and the presence of an obstructive lesion in the pulmonaryvenous channel.^3^ Patients with unobstructed TAPVC are usually asymptomatic at birth; however, by the first few weeks of life, they will present with evidence of a large left-to-right shunt, cardiomegaly, failure to thrive, mild cyanosis, and cardiorespiratory failure. Children with obstructed TAPVC will present with respiratory problems, hypoxia, pulmonary hypertension, rapid progression to cardiorespiratory failure, and respiratory-distress-syndrome-like pattern.^1^^, ^^2^ These children require urgent repair, while those with unobstructed TAPVC can have a more elective correction.^2^

The surgical repair of supracardiac TAPVC has undergone several modifications, and most surgeons prefer to ligate the vertical vein to prevent residual left-to-right shunting. However, with smaller left-sided chambers and non-compliant left atrium, an unligated vertical vein may improve survival by providing a transitory decompression of the right side of the heart for postoperative pulmonary hypertension crises.^3^ The unligated vertical vein has been reported to atrophy spontaneously, but there have been reports where the unligated vertical vein remains patent and results in significant symptoms due to a left-to-right shunt.^4^ Such patients can be candidates for percutaneous angiographic closure by device. The transcatheter closure of the unligated vertical vein with a ductus occluder in supracardiac TAPVC has been reported in a few instances.^5^^, ^^6^

In this study, we report the transcatheter closure of a partially ligated vertical vein using the vascular plug device in our patients with previous repair of supracardiac TAPVC.

## Case Report

***Patient 1***

A 2-year-old (10 kg) infant with unobstructed supracardiac TAPVC underwent surgical correction at Faghihi Hospital, Shiraz, Iran. Preoperative echocardiography showed that the pulmonary venous channel drained through a vertical vein into the innominate vein and thereafterinto the superior vena cava. During surgery, the vertical vein was partially banded due tohigh pulmonary artery pressure. After discharge, the patient presented with a gradual decrease in pulmonary pressure and desirable weight gain. In echocardiography, return of the pulmonary veins to the left atrium, an open vertical vein with an ascending flow into the innominate vein, and mildly dilated right atrium and right ventricle were detected. Thus, the patient wascandidated for the percutaneous closure of the vertical vein by device.

Cardiac catheterization was performed 23months after the surgical correction. Through a 5-Fr sheath via the femoral vein, a 5-Fr NIH catheter was advanced to the branch pulmonary arteries. Injection of contrast showed that the pulmonary veins drainedinto the left atrium and flowedinto the innominate vein through a patent and dilated vertical vein. In addition, a 5-Fr right Judkins catheter was advanced over a 0.035-in guide wire through the superior vena cava and theinnominate vein into the vertical vein. The mean pressure in the vertical vein was 10mmHg, mean right atrial pressure was 6 mmHg, right ventricular pressure was 40 mmHg, and mean pulmonary artery pressure was 17 mmHg. 

**Figure 1:**
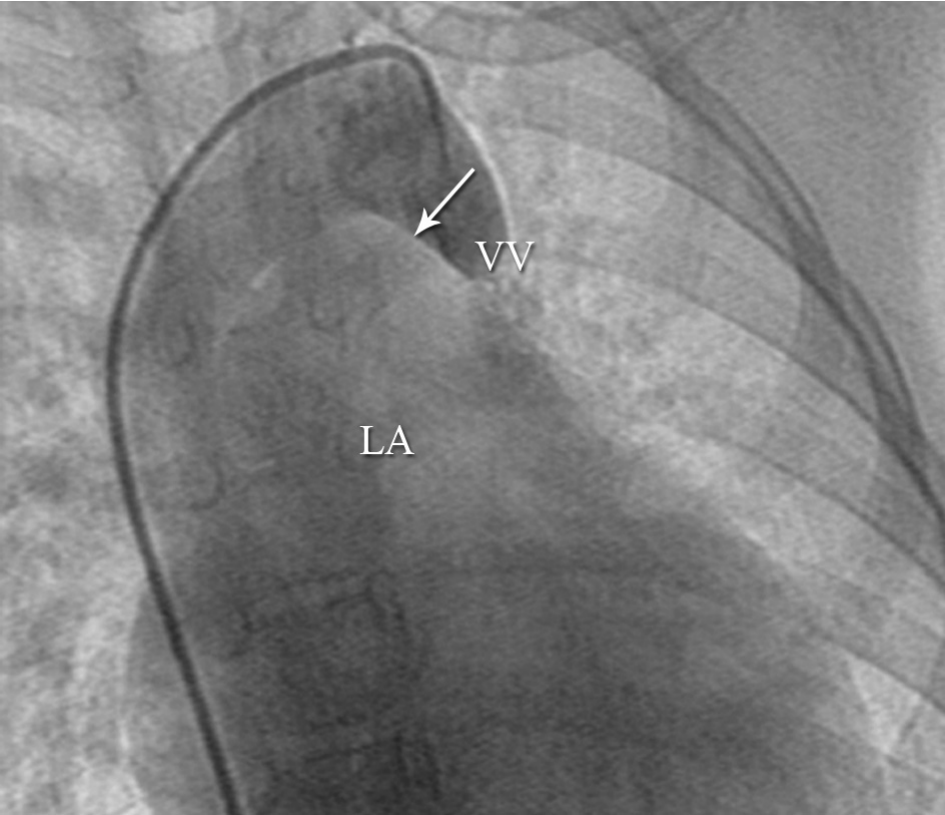
Angiographic imaging of Case # 1 in the anteroposterior projection, revealing the passage of the contrast dye (arrow) from the innominate vein through a patent vertical veininto the left atrium.

LA, Left atrium; Inno, Innominate vein; RA, Right atrium; SVC, Superior vena cava; VV, Vertical vein; LA, Left atrium

**Figure 2:**
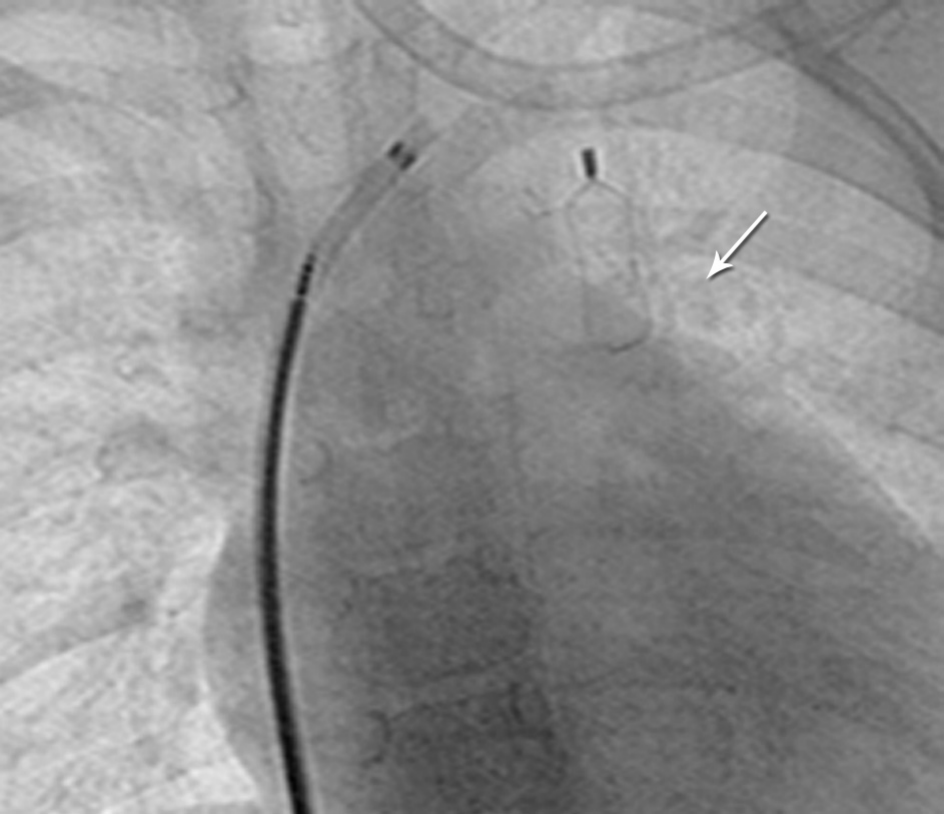
Angiogram in the anteroposterior view in Case #1 after the occlusion of the vertical vein with an Amplatzer vascular plug. The arrow shows vascular plug type 1.

**Figure 3:**
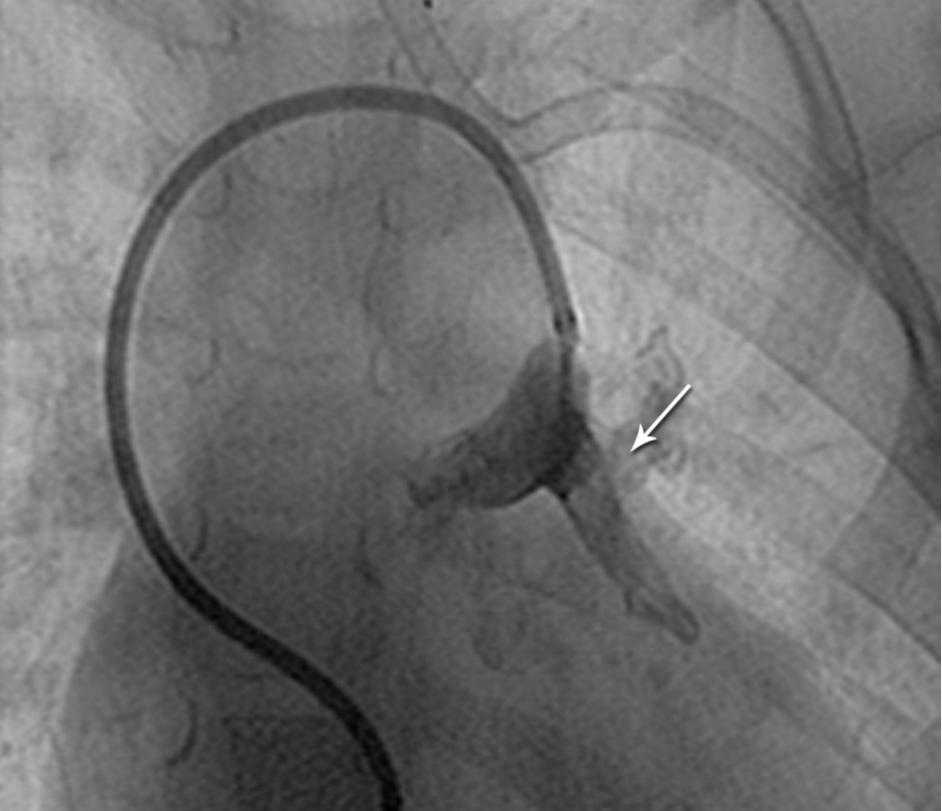
Angiogramin the anteroposterior view of Case # 2, demonstrating a catheter injection in the proximal portion of the vertical vein (arrow).

Angiography of the vertical vein demonstrated that it drained into the innominate vein (Figure 1). In the anteroposterior view, the diameter of the stenotic part of the vertical vein was 7 mm. Subsequently, the ascending vein was occluded with a 10-mm vascular plug type I (Figure 2). Post-occlusion angiography in the ascending vertical vein showed a minimal residual flow (Figure 3). During a 2-year follow-up period, the infant showed no increase inpulmonary artery pressure.

Inno, Innominate vein; RA, Right atrium; SVC, Superior vena cava; VV, Vertical vein; LA, Left atrium

**Figure 4:**
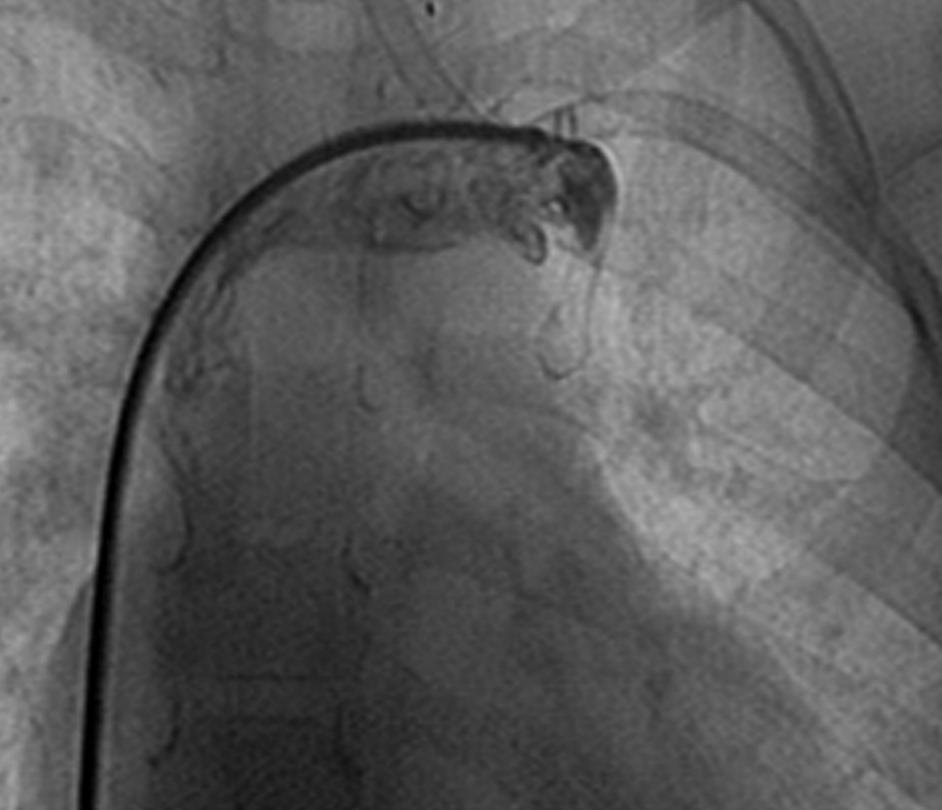
Angiogram in the anteroposterior view in Case # 2 after the device closure of the unligated vertical vein, demonstrating no flow through the vein

Inno, Innominate vein; RA, Right atrium; SVC, Superior vena cava; VV, Vertical vein; LA, Left atrium

***Patient 2***

A 5-year-old (15 kg) infant with unobstructed supracardiac TAPVC underwent surgical repair and the vertical vein was partially ligated as was described in the previous case.

Postoperative findings via echocardiography showed a gradual decrease in pulmonary pressure and good growth. In cardiac catheterization, which was performed 14 months after the primary repair, the mean right atrial pressure was 8 mmHg, right ventricular pressure was 30 mmHg, and mean pulmonary artery pressure was 21 mmHg. The stenotic part was 6 mm, and the vertical vein was successfully closed with an 8-mm vascular plug type 1 (Figure 4). The patient had a suitable condition during a 2-year follow-up period. 

## Discussion

TAPVC is categorized into four types defined by the site ofthe entry of the anomalous connection. The most common type is the supracardiac type, followed by the infracardiac type, while the cardiac and mixed types are relatively uncommon.^7^ Nowadays, the mortality rate has decreased due to the recent modifications in the surgical repair; i.e., leaving the vertical vein open at the time of TAPVC correction inpatients with a significantly small and non-compliant left atrium.^4^^, ^^7^^, ^^8^

Maully J. Shah et al.^5^ suggested that leaving the vertical vein patent at the time of TAPVC repair may afford better postoperative conditions by providing a temporary pop-off for pulmonary hypertensive crisis.^5^ Since the patient's venous pathway can continue to function as a conduit for a significant left-to-right shunt, this overload increases the work of the right heart chambers and leads to symptoms. Consequently, the transcatheter closure of an open vertical vein has been recommended. Few instances havealso been reported for the device closure of the vertical vein. N. Wilson^6^ and HG. Schneider et al.^9^ reported the successful closure of unligated vertical veins usingductusoccluders in 2 patients with supracardiac TAPVC. Also, D. Kobayashi et al.^4^ reported the successful transcatheter closure of a persistent patent vertical vein usingthe Amplatzer vascular plug device and a Gianturco coil.

In the present study, we described 2 patients with partially ligated vertical veins who showed a gradual decrease in pulmonary pressure and improvement of cardiacas well as pulmonary function. The transcatheter closure of the open vertical veins with vascular plugs type I was done successfully and no adverse event was observed during a 2-year follow-up period. Therefore, this procedure could be a useful method for the closure of a persistent patent vertical vein in surgically corrected TAPVC.

## Conclusion

The transcatheter closure of a partially ligated vertical vein using the vascular plug device in patients with previous repair of supracardiac TAPVC could be a useful and safe management modality for the prevention of subsequent complications due to residual left-to-right shunting.

## Notes:

***This paper should be cited as: ***Amoozgar H, Ahmadipoor M, Amirghofran AA. Transcatheter Closure of Partially Ligated Vertical Vein after Surgical Correction of Supracardiac Total Anomalous Pulmonary Venous Connection. J The Univ Heart Ctr 2015;10(3):152-155.
